# Determination of Methemoglobin in Hemoglobin Submicron Particles Using NMR Relaxometry

**DOI:** 10.3390/ijms21238978

**Published:** 2020-11-26

**Authors:** Waraporn Kaewprayoon, Nittiya Suwannasom, Chiraphat Kloypan, Axel Steffen, Yu Xiong, Eyk Schellenberger, Axel Pruß, Radostina Georgieva, Hans Bäumler

**Affiliations:** 1 Charité-Universitätsmedizin Berlin, Institute of Transfusion Medicine, 10117 Berlin, Germany; waraporn.kpy@gmail.com (W.K.); nittiya.su@up.ac.th (N.S.); chiraphat.kl@up.ac.th (C.K.); Axel.steffen@charite.de (A.S.); Yu.Xiong@charite.de (Y.X.); axel.pruss@charite.de (A.P.); radostina.georgieva@charite.de (R.G.); 2 Department of Pharmacy, Payap University, Chiang Mai 50000, Thailand; 3 Division of Biochemistry and Nutrition, School of Medical Sciences, University of Phayao, Phayao 56000, Thailand; 4 Division of Clinical Immunology and Transfusion Sciences, School of Allied Health Sciences, University of Phayao, Phayao 56000, Thailand; 5 Charité-Universitätsmedizin Berlin, Institute of Radiology and Children Radiology, 10117 Berlin, Germany; eyk.schellenberger@charite.de; 6 Department of Medical Physics, Biophysics and Radiology, Faculty of Medicine, Trakia University, 6000 Stara Zagora, Bulgaria

**Keywords:** methemoglobin, hemoglobin microparticles (Hb-MP), ^1^H_2_O NMR-relaxometry, hemoglobin-based oxygen carriers

## Abstract

Methemoglobin (MetHb) is a hemoglobin (Hb) derivative with the heme iron in ferric state (Fe^3+^), unable to deliver oxygen. Quantification of methemoglobin is a very important diagnostic parameter in hypoxia. Recently, novel hemoglobin microparticles (Hb-MP) with a narrow size distribution around 700 nm, consisting of cross-linked Hb were proposed as artificial oxygen carriers. The cross-linking of Hb by glutaraldehyde (GA) generates a certain amount of MetHb. Due to the strong light scattering, quantitative determination of MetHb in Hb-MP suspensions by common spectrophotometry is not possible. Here, we demonstrate that ^1^H_2_O NMR relaxometry is a perfect tool for direct measurement of total Hb and MetHb concentrations in Hb-MP samples. The longitudinal relaxation rate 1/T_1_ shows a linear increase with increasing MetHb concentration, whereas the transverse relaxation rate 1/T_2_ linearly increases with the total Hb concentration. In both linear regressions the determination coefficient (R^2^) is higher than 0.99. The method does not require time-consuming pretreatment or digestion of the particles and is not impaired by light scattering. Therefore, it can be established as the method of choice for the quality control of Hb-MP and similar hemoglobin-based oxygen carriers in the future.

## 1. Introduction

Hemoglobin (Hb), the main component of red blood cells (RBC), is an iron-containing oxygen-transporting metalloprotein. It contains four heme groups each incorporating an iron atom in the ferrous state (Fe^2+^), which can reversibly bind molecular oxygen (O_2_), resulting in two states of Hb—the oxygenated Hb (OxyHb) and the deoxygenated Hb (DeoxyHb). In this way, RBC are loaded with O_2_ in the lung (OxyHb) and O_2_ is delivered to the organs/tissue (DeoxyHb) [1,2]. In contrast to these two Hb-states, Hb derivatives are altered forms of hemoglobin, which are formed by the combination of different iron ligands in the heme or by the oxidation state of the iron. After oxidation, the iron in the heme group changes to the ferric state (Fe^3+^) and Hb becomes methemoglobin (MetHb). MetHb cannot bind or carry oxygen molecules since the sixth coordination position of iron is bound either to a water molecule or a hydroxyl group [3,4]. Furthermore, the presence of MetHb increases the affinity of the remaining Hb or heme groups to oxygen, which makes the oxygen release in the tissues more difficult [4,5]. Normally one to two percent of a person’s Hb is MetHb [5]. A higher percentage, can be of genetic origin or caused by exposure to various chemicals [3,6]. Depending on the level of MetHb in the blood, it can cause health problems known as methemoglobinemia. Therefore, the MetHb concentration in blood is a very important diagnostic parameter in cases of hypoxia.

One of common methods of hemoglobin analysis in clinical laboratories are based on the so called multiple wavelength spectrophotometry (CO-oximetry) [7,8,9,10], which exploits the differences in the absorption spectra of OxyHb, DeoxyHb, and MetHb. Before measurement, Hb is released from the RBCs by hemolysis to avoid the light scattering by the cells.

Recently, novel hemoglobin microparticles (Hb-MP) have been developed and proposed as artificial oxygen carriers. The fabrication procedure was published under the abbreviation CCD-technique (Co-precipitation-Crosslinking-Dissolution) [11,12,13] that reflects the three main preparation steps: co-precipitation of Hb with MnCO_3_, crosslinking of Hb and dissolution of the MnCO_3_. The resulting particles consist of cross-linked Hb and show a narrow size distribution around 700 nm. The particles were investigated spectrophotometrically to demonstrate their oxygenation and deoxygenation [11,14]. However, Hb-MP cannot be lysed like RBC and a quantitative determination of OxyHb, DeoxyHb, and MetHb by multiple-wavelength spectrophotometry is not possible due to the strong light scattering of the Hb-MP suspensions. It is known, that the cross-linking of Hb by glutaraldehyde (GA) causes a certain amount of MetHb to arise [15]. Therefore, a quantitative measurement of the MetHb content of the Hb-MP is very important in view of their potential application as an artificial blood substitute and alternatives to spectrophotometric measurements are needed.

In previous studies, the oxygen carrying capacity of the Hb-MP was used for the indirect determination of dyshemoglobin or nonfunctional hemoglobin. The oxygen released after oxidation of the Hb by cyanide was directly measured and related to the amount of functional Hb in the Hb-MP [12,16]. The difference between total Hb and functional Hb equals nonfunctional hemoglobin. However, this method does not allow a direct measurement of MetHb and presupposes 100% oxygenation of the Hb in the Hb-MP.

The water proton nuclear magnetic resonance (^1^H_2_O NMR) relaxometry could be a suitable method that can be used for quantification of the MetHb concentration directly by measuring the oxidative state of iron in the Hb-MP. The different oxidation state of the iron in MetHb means that MetHb has a different electronic configuration and, therefore, different paramagnetic properties compared to that of Hb [17]. This difference can be detected using NMR spectroscopy by measuring the ^1^H_2_O NMR longitudinal relaxation time (T_1_) and the transverse relaxation time (T_2_) or the corresponding relaxation rates (1/T_1_ and 1/T_2_). Previous studies on methemoglobin concentrations in blood samples have shown comparable values obtained by NMR and multiples-wavelength spectrophotometry [18,19] but the NMR method is not influenced by light scattering and does not require lysis of RBC. Additionally, NMR provides information on the molecular environment and therefore it would allow to investigate the influence of the GA crosslinking on the molecular structure of Hb in the Hb-MP.

The aim of this study was to demonstrate that the ^1^H_2_O NMR relaxation rates can be used to determine the total Hb as well as MetHb content of Hb-MP, because multiple wavelength spectrophotometry cannot be applied. Therefore, the ^1^H_2_O NMR relaxation times of Hb in Hb-solutions and in vital RBC were measured at various MetHb concentrations to investigate the influence of different surroundings on their relaxation properties. The influence of the GA crosslinking on the ^1^H_2_O NMR relaxation rates was investigated using GA cross-linked RBC and GA HSA-MP. Finally, Hb-MP suspensions with different MetHb contents were prepared and the ^1^H_2_O NMR relaxation rates were measured.

## 2. Results and Discussion

### 2.1. NMR of Human Hb/MetHb Solutions and RBCs/MetHb-RBC Suspensions

**Figure 1:**
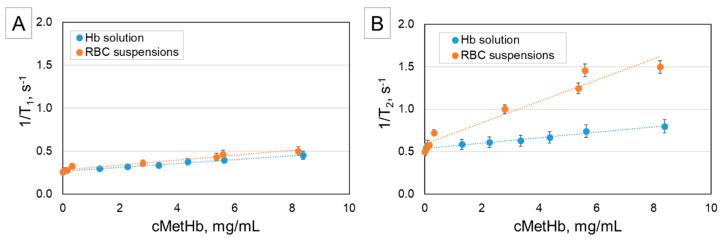
Relaxation rates 1/T_1_ (**A**) and 1/T_2_ (**B**) of Hb solutions and of RBC suspension as a function of the MetHb concentration (measured at proton frequency 40 MHz and temperature 37 °C). The values represent means and standard deviations of N = 6 samples. The slopes of 1/T_1_ for Hb solutions (0.0226) as well as for RBC suspensions (0.0287) are similar, but for 1/T_2_ the slopes are different (0.0317 for Hb solutions; 0.1254 for RBC suspensions).

Figure 1 shows the relaxation rates 1/T_1_ and 1/T_2_ of Hb solutions and suspensions of intact RBCs depending on the MetHb concentration. It can be seen that both, the longitudinal as well as the transverse relaxation accelerate with increasing MetHb concentration. The increase of 1/T_1_ with increasing MetHb concentration is roughly equivalent in Hb solutions and suspensions of intact RBCs (Figure 1A), whereas 1/T_2_ increases much faster with the MetHb concentration in RBC suspensions (Figure 1B). This is in agreement with the results obtained by other groups [20,21,22,23].

The paramagnetic ^1^H_2_O NMR relaxation of MetHb includes complex mechanisms with contributions from both the inner shell (bound water molecules) and outer shell (diffusional water molecules) [24,25,26]. Since all samples were prepared and processed under normal atmospheric conditions, OxyHb was the mean compound in the vital RBC obtained from the donor’s blood. The electron configurations of the iron in OxyHb and MetHb are completely different. In OxyHb all electrons of the outer shell remain paired on lower energetic levels due to the strong ligand field of oxygen, whereas MetHb has five unpaired electrons. Additionally, the transformation of Hb to MetHb leads to a conformational change of the protein that opens the hydrophobic pocket normally protecting the iron in the heme from interaction with water. The unpaired electrons coupled with their accessibility by the water are behind the paramagnetic properties of MetHb and are directly responsible for the increase of 1/T_1_ by proton-electron dipole-dipole interaction.

The direct paramagnetic effects of MetHb contribute also to 1/T_2_. This is visible in the slight increase of the transverse relaxation rate of Hb solutions with increasing MetHb content (Figure 1B). The much faster transverse relaxation in the intact RBC suspensions is caused by a diffusional mechanism. Here, the paramagnetic MetHb is concentrated inside the RBC and leads to a great difference of the magnetic susceptibility between the cell interior and the surrounding solution. Consequently, magnetic field gradients are generated within and around the RBC, influencing the protons of the water molecules diffusing across these gradients and causing T_2_ proton relaxation enhancement (PRE) [17]. Additionally, at the same total amount of Hb in the samples the concentration of Hb in the RBC is much higher than that in the Hb solutions impeding the Brownian movement of water. This so-called “water of hydration effect” also influences the transverse relaxation time [27].

Since bovine Hb is used for the fabrication of Hb-MP, the ^1^H_2_O NMR relaxation behavior of bovine Hb solutions and bovine RBC suspensions were compared in one experiment and it was confirmed that they are not different (Supporting Information, Figure S2).

### 2.2. NMR of RBCs Treated with GA (Influence of Crosslinking)

In the fabrication of Hb-MP a cross-linking step by GA is involved. GA randomly reacts with two amino groups of the proteins causing intramolecular or intermolecular bridges between the peptide chains [28,29]. It is known, that such chemical modifications lead to altered ^1^H_2_O relaxation times due to changes in molecular weight, viscosity, chain flexibility, etc. [30]. In the case of Hb the reaction with GA is also connected with oxidation of the heme iron and formation of MetHb to some extent, which mainly depends on the applied GA concentration. Therefore, the influence of GA cross-linking on the ^1^H_2_O relaxation rates was investigated in suspensions of RBCs cross-linked with different GA concentrations.

**Figure 2:**
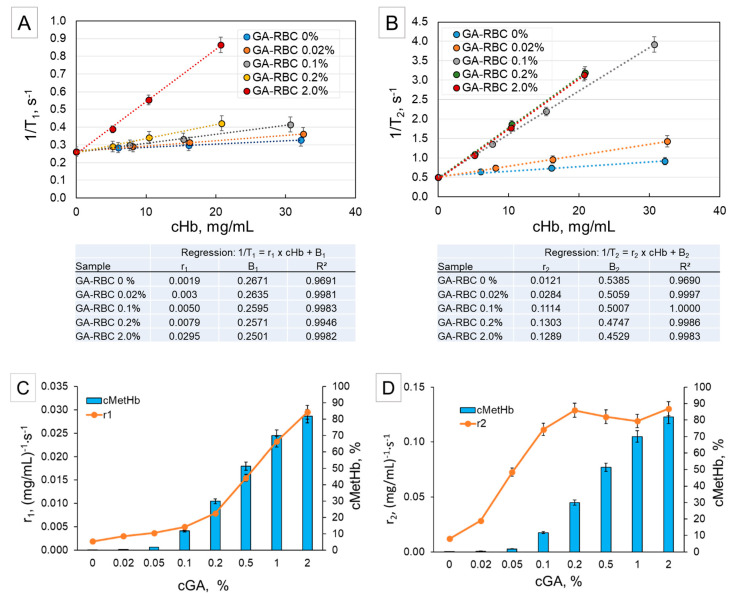
(**A**) Longitudinal relaxation rates 1/T_1_ and (**B**) transverse relaxation rates 1/T_2_ of RBC suspensions treated with different GA concentrations depending on the total hemoglobin concentration. The results are fitted by a linear regression with the parameters shown in the tables below the corresponding graphs with r_1_ and r_2_ representing the slope (or the so called relaxivities), respectively. (**C**,**D**) Correlation between GA concentration, methemoglobin concentration (blue columns), and relaxivities r_1_ and r_2_ (orange curves), respectively. All measurements were performed at a proton frequency 40 MHz and a preset temperature of 37 °C.

The results of these experiments are summarized in Figure 2. The relaxation rates 1/T_1_ and 1/T_2_ (Figure 2A,B, respectively) of RBC suspensions cross-linked with four different GA concentrations are plotted against the total Hb concentration (cHb) in the samples. For comparison, the relaxation rates of native RBC (0% GA) are also shown. It can be seen that both relaxation rates have a linear dependency. The slopes of 1/T_1_ and 1/T_2_ calculated by regression analysis differ by a factor of 10 (tables in Figure 2), and represent r_1_ and r_2_, respectively, the so-called relaxivity. The slope of the longitudinal relaxation rate 1/T_1_ increases with increasing GA concentration used for cross-linking, whereas the slope of the transverse relaxation rate 1T_2_ reaches a maximum at a GA concentration around 0.1–0.2% and does not increase further. These relaxivities, r_1_ and r_2_, are presented in correlation with the GA concentration and the corresponding MetHb concentration on Figure 2C,D, respectively. It clearly demonstrates that r_1_ or the longitudinal relaxation is dominated by the paramagnetic effect and therefore directly depends on the increasing MetHb (or Fe^3+^) concentration. In contrast, r_2_ increases rapidly at low GA-concentrations and reaches a plateau at 0.1% GA.

T_1_ decreases with increasing GA concentration because the MetHb concentration increases correspondingly and T_1_ mainly reflects paramagnetic effects. In contrast, T_2_ decreases dramatically at very low GA-concentrations where the MetHb formation is lower than 20%. This is due to the rapid increase of the degree of cross-linking already at low GA concentrations. At the same time, the dependency of T_2_ on the paramagnetic effect of MetHb is very weak. This becomes evident at high GA concentrations, where MetHb increases dramatically but T_2_ (and respectively r_2_) are constant. When the cross-linking of all proteins is complete, r_2_ reaches a steady state and the paramagnetic effect of relaxation is dominated by the effect of the protein crosslinking [30,31,32].

### 2.3. NMR of HSA-MP, Hb-MP, and MetHb-MP

All particles used in this study were prepared by the CCD-technique [14] and GA was used for cross-linking (Table 1). The GA concentration applied for the fabrication of Hb-MP was 0.02%, as low as possible in order to minimize the oxidation of the heme iron and the generation of MetHb. HSA-MP were prepared with three concentrations of GA, including 0.02%. These particles were used as a control of iron free protein particles to observe the effect of the cross-linking of the proteins.

**Figure 3:**
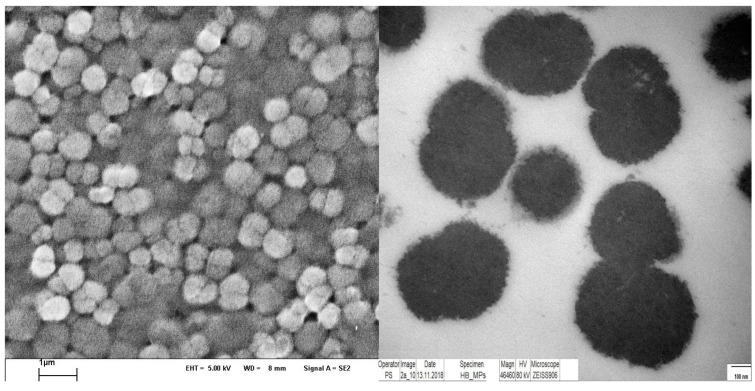
Scanning electron micrograph (**left**) of Hb-MP as overview showing the uniform size distribution of the particles in the submicron size range. Transmission electron micrograph of Hb-MP at higher magnification (**right**).

The size of all particle types was in the range of 700–850 nm. The narrow size distribution and the peanut like shape is visible in the scanning as well as in the transmission electron micrograms (Figure 3). Hb-MP and MetHb-MP contained 22–24 mg/mL Hb at a packed particle volume (PPV) concentration of 20%. This is in agreement with previously reported values for Hb-MP [33]. The treatment with NaNO_2_ resulted in particles with more than 96% MetHb (for simplicity these particles are referred as MetHb-MP). The content of OxyHb in the Hb-MP and MetHb-MP was determined by the oxygen release method and was roughly 50% of the total Hb in the Hb-MP and less than 4% in the MetHb-MP.

**Figure 4:**
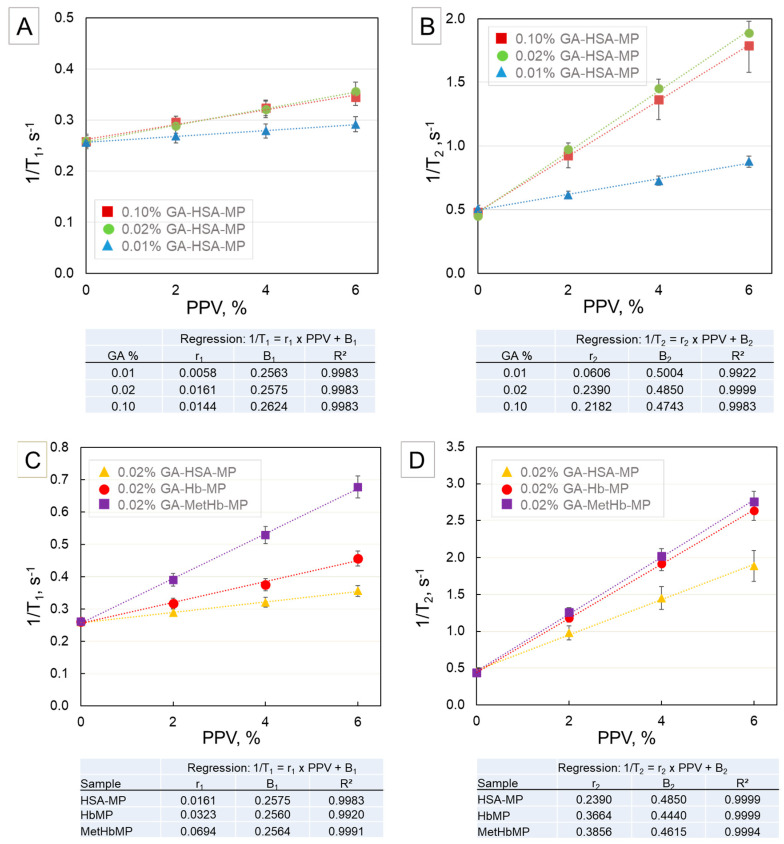
Relaxation rates of HSA-MP suspensions prepared with different concentrations of GA depending on the particle volume concentration (PPV). (**A**) Longitudinal relaxation rate (1/T_1_) and (**B**) transverse relaxation rate (1/T_2_). Comparison of (**C**) longitudinal relaxation rate (1/T_1_) and (**D**) transverse relaxation rate (1/T_2_) of HSA-MP, Hb-MP, and MetHb-MP suspensions, prepared with 0.02% GA depending on the particle volume concentration (PPV). The parameters applied for the linear regressions are shown in the tables below the corresponding graphs. Results were obtained at a proton frequency 40 MHz and temperature 37 °C.

The graphs in Figure 4A,B show the longitudinal relaxation rate (1/T_1_) and the transverse relaxation rate (1/T_2_), respectively, of HSA-MP suspensions prepared with different concentrations of GA depending on the particle volume concentration (packed particle volume, PPV). In all samples, the relaxation rates linearly increase with increasing particle concentration. Since HSA-MP do not contain any paramagnetic element, the increase of the relaxation rates is only due to the increase of the protein amount in the samples. The slopes of the linear regressions (or relaxivities, r_1_ and r_2_) obtained for the samples prepared with 0.02% and 0.1% GA are practically equal. However, they are much higher than that obtained for the sample with 0.01% GA. This means that already at a GA-concentration of 0.02%, the formation of the protein cross-linked network is completed and therefore higher GA concentration does not contribute to structural changes in the molecular structure of the particles.

Hb-MP and MetHb-MP were prepared with 0.02% GA and presumably, the protein cross-linking is completed and should not influence the relaxation rates. In Figure 4C,D the 1/T_1_ and 1/T_2_ of HSA-MP, Hb-MP, and MetHb-MP suspensions, prepared with 0.02% GA depending on the particle volume concentration (PPV) are presented. The results are fitted by a linear regression with the parameters shown in the tables below the corresponding graphs. Both relaxivities are enhanced in comparison with the respective relaxivities of the HSA-MP, which corresponds to the presence of the heme iron. The longitudinal relaxivity, r_1_, of MetHb-MP is twice as high as that of the Hb-MP because more than 96% of the iron is in the paramagnetic Fe^3+^ state. In contrast, the transverse relaxivity, r_2_, of MetHb-MP is not very different from that of the Hb-MP, because the cross-linking of the proteins is completed and the paramagnetic effect of MetHb has no influence on T_2_. It has to be noted that the GA concentration necessary to complete the protein cross-linking in the particles is roughly five times lower than that needed to achieve the same effect in RBC (0.02% for all investigated particles and 0.1% for RBC). However, the exact calculation of the ratio of GA to protein in both cases is difficult since both systems are very different. Additionally, the permeability of the RBC membrane and the cross-linking of the membrane proteins may contribute to this difference.

### 2.4. NMR as a Tool for Determination of Total Hb and MetHb in Hb-MP

We mixed Hb-MP and MetHb-MP suspensions (prepared with 0.02% GA) at the ratios 100/0, 80/20, 60/40, 40/60, 20/80, and 0/100 and diluted them with PBS to obtain suspensions with final PPV of 2%, 4%, and 6%. The total Hb concentration (cHb) and the MetHb concentration (cMetHb) in the mixtures were calculated using the data for the Hb-MP and the MetHb-MP in Table 1.

**Figure 5:**
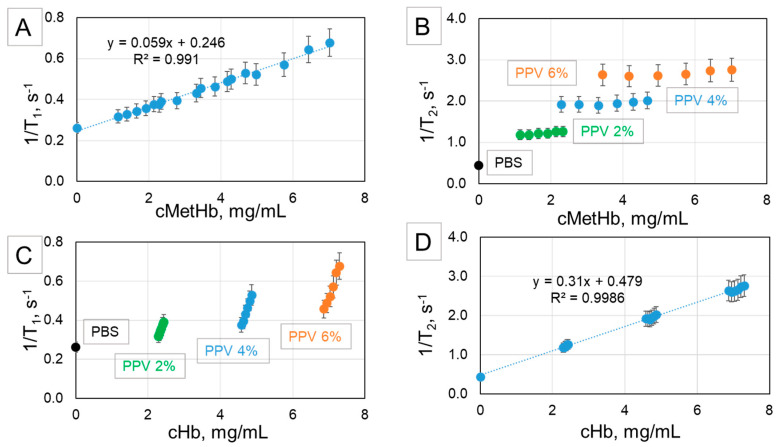
Relaxation rates of Hb-MP/MetHb-MP mixed suspensions prepared with 0.02% GA. Hb-MP and MetHb-MP are mixed at ratios 100/0, 80/20, 60/40, 40/60, 20/80, and 0/100 and diluted to obtain suspensions with PPV of 2%, 4%, and 6%. (**A**) Longitudinal relaxation rate (1/T_1_) and (**B**) transverse relaxation rate (1/T_2_) depending on the MetHb concentration in the suspensions. (**C**) Longitudinal relaxation rate (1/T_1_) and (**D**) transverse relaxation rate (1/T_2_) depending on the total Hb concentration. The measurements were performed at a proton frequency of 40 MHz and a preset temperature of 37 °C.

The obtained relaxation rates 1/T_1_ and 1/T_2_ of all mixtures and dilutions were plotted against cMetHb (Figure 5A,B, respectively) and cHb (Figure 5C,D, respectively). It can be seen that the longitudinal relaxation rate 1/T_1_ shows a linear increase with increasing cMetHb (Figure 5A), whereas 1/T_2_ linearly depends on cHb (Figure 5D). In both cases the data are fitted by a linear regression with a determination coefficient (R^2^) higher than 0.99.

As expected, 1/T_2_ is not influenced much by the MetHb content. As seen in Figure 5B, the values for the transverse relaxation of mixed suspensions with the same PPV are not different. In contrast, the longitudinal relaxation rate of mixed suspensions with the same PPV is strongly dependent on the ratio Hb-MP/MetHb-MP as it is demonstrated in Figure 5C, where 1/T_1_ is plotted against the total Hb concentration.

In conclusion, we could confirm the linear increase of 1/T_1_ and 1/T_2_ as a function of the MetHb concentration in Hb solutions and RBC suspensions as already shown by other studies. The much faster increase of 1/T_2_ in suspensions of intact RBC is explained by the unequal distribution of Hb/MetHb between cell interior and surrounding solution. Consequently, the generated magnetic field gradients within and around the RBC lead to relaxation enhancement caused by the diffusion of water across them. The so-called “water of hydration effect” caused by the impeded Brownian movement of water contributes also to the faster transverse relaxation in RBC suspensions.

The results obtained for suspensions of RBCs cross-linked with different GA concentrations revealed that the longitudinal and the transverse relaxation behavior deliver information on the two different aspects of the GA cross-linking: (i) degree of iron oxidation and MetHb generation via longitudinal relaxivity r_1_ and (ii) degree of cross-linking and protein network formation via transverse relaxivity r_2_, respectively. Additionally, the measurements of HSA-MP confirmed that the degree of network formation mainly influences the transversal relaxation. These results were very important for the interpretation of the data obtained for Hb-MP.

Finally, the measurements performed with Hb-MP and MetHb-MP confirmed that ^1^H_2_O NMR is a perfect tool for investigation of Hb-MP samples by direct measurement of their total Hb (using the transverse relaxation rate, 1/T_2_) and MetHb (using the longitudinal relaxation rate 1/T_1_). The method does not require time-consuming pretreatment or digestion of the particles and is not impaired by light scattering like spectroscopic methods. Therefore, it can be established as the method of choice for the quality control of Hb-MP and similar hemoglobin based oxygen carriers in the future.

## 3. Materials and Methods

### 3.1. Materials

Fresh human blood samples were collected from healthy volunteers in accordance with the transfusion law of Germany. Informed consent was obtained from all donors in written form. The use of donor blood samples for scientific purposes was approved by the ethics committee of Charité-Universitätsmedizin Berlin (# EA1/137/14). The blood was withdrawn into K2 EDTA (1.8 mg/mL, EDTA) blood tubes, Becton Dickinson (BD), Heidelberg, Germany.

Fresh bovine whole blood (EDTA-anticoagulated) was obtained from Biophyll GmbH, Dietersburg, Germany.

Phosphate buffered saline pH 7.4 (PBS pH 7.4) was purchased from Fisher bioreagents^®^, Fisher Scientific, Hampton, NH, USA. Sodium nitrite (NaNO_2_), glutaraldehyde 25% (GA), manganese chloride (MnCl_2_), sodium carbonate (Na_2_CO_3_), disodium ethylenediaminetetraacetate (Na_2_-EDTA), potassium ferricyanide (K_3_[Fe(CN)_6_]), sodium chloride (NaCl), Triton-X-100, were purchased from Sigma-Aldrich GmbH, Darmstadt, Germany.

### 3.2. Preparation of Vital RBC, GA-Treated RBC (GA-RBC), and Sodium Nitrite Treated RBC (MetHb-RBC) Suspensions

Immediately after blood collection, tubes were slowly agitated to ensure an appropriate mixing of anticoagulant. The blood samples were centrifuged at 2000× *g*, 10 min, 4 °C to remove plasma and buffy coat and the isolated RBCs were washed three times with PBS pH 7.4. The washed RBCs were resuspended in PBS pH 7.4, the hematocrit (Hct) was measured and adjusted to 10% (centrifugation at 20,000× *g*, 10 min).

RBC suspensions with 10% Hct were incubated with 0.02%, 0.05%, 0.1%, 0.2%, 0.5%, 1.0%, and 2.0% GA, respectively (room temperature 22 °C, 1 h). After incubation, the GA-RBCs were washed five times with PBS pH 7.4 (centrifugation at 2000× *g*, 10 min, 4 °C) to remove unbound GA and finally the initial volume of the suspension was reconstituted with PBS pH 7.4. The total iron content (cFe) of the GA-RBC suspensions was measured by inductively coupled plasma optical emission spectroscopy (ICP-OES; Thermo, iCAP 6300 Duo, Thermo Fisher Scientific GmbH, Berlin, Germany). Before measurement the GA-RBCs were dissolved with 6 M HCl. The values obtained for the total iron were used to calculate the total Hb concentration in the GA-RBC samples. The cOxyHb concentration was determined indirectly by the oxygen release method as described below. The MetHb concentration was calculated as the difference between total Hb and OxyHb.

Sodium nitrite (NaNO_2_) reacts in aqueous millieu with Fe^2+^ of Hb oxidizing it to Fe^3+^ and therefore converting Hb to MetHb [34,35]. Native RBC suspensions (10% Hct in PBS (pH 7.4)) were incubated with 5 mM NaNO_2_ under gentle mixing at room temperature (22 °C) for 30 min and were thereafter stored overnight at 4 °C. The MetHb-RBCs were washed three times with PBS pH 7.4 at 2000× *g*, 10 min, 4 °C and finally the RBC concentration was adjusted to 10% Hct with PBS, pH 7.4.

### 3.3. Preparation of Hb and MetHb Solutions

Hb solutions were prepared by lysing the RBCs with diluted PBS pH 7.4 at 100 mOsmol/kg. The lysed RBCs were centrifuged at 10,000× *g*, 10 min, 4 °C to eliminate the cell membranes. The supernatant was collected and filtered through a membrane filter (pore size 0.2 µm; Carl Roth GmbH, Karlsruhe, Germany). Total Hb concentration (cHb) was measured using ABL700 (Radiometer^®^, Copenhagen, Denmark) and adjusted to 30 mg/mL by dilution with PBS pH 7.4.

MetHb solutions were prepared lysing MetHb-RBCs with diluted PBS (pH 7.4 at 100 mOsmol/kg) as described for the Hb solution above.

### 3.4. Determination of Oxyhemoglobin in RBC Suspensions and Hb Solutions

Two approaches were applied for the determination of oxyhemoglobin (OxyHb) in RBC suspensions and Hb solutions—a standard method using a blood gas analyzer (ABL700, Radiometer^®^, Copenhagen, Denmark) and an indirect method based on measurement of the oxygen release after oxidation of the iron in the heme by K_3_[Fe(CN)_6_] [36,37]. The results delivered by the two methods for Hb solutions and RBC suspensions correlated very well with each other (Supporting Information, Figure S1).

The optical system of ABL700 includes a 128-wavelength spectrophotometer connected via an optical fiber with a measuring chamber in which the blood sample is hemolyzed by ultra-sonication. The measurements were conducted in the wavelength range 478–672 nm. The concentration of each compound is determined using the following equation:

(1) $$c_{y}=\overset{128}{\underset{y=1}{\sum }}K_{y}^{\lambda_{n}}\;A_{total}^{\lambda_{n}}$$

where $A_{total}^{\lambda_{n}}$ is the total absorption of the sample measured at a certain wavelength *λ_n_*, $K_{y}^{\lambda_{n}}$ are the constants specific to each compound at this wavelength.

In such a way the blood gas analyzer delivers data on the total Hb concentration (cHb) and percentage of hemoglobin derivatives (mainly OxyHb and MetHb).

The oxygen release measurement was performed using a miniaturized optical needle-type oxygen sensor (oxygen microsensor NTH-PSt7, PreSens, Regensburg, Germany) connected with a portable oxygen meter with data logging (Microx 4, PreSens—Precision Sensing GmbH, Germany). A total of 100 µL of Hb solution (30 mg/mL) or 100 µL of RBC suspension (Hct 10%) were added into 900 µL 0.1% Triton-X100 solution in PBS pH 7.4 and were stirred for 5 min. After stabilization of the measured value for the oxygen concentration in the solution with one measuring point per second, 50 µL of 10% K_3_[Fe(CN)_6_] were added and the increase of the oxygen concentration in the solution is detected. The amount of OxyHb in the solutions can be calculated using Equations (2) and (3):

(2) $$mO_{2}\left(µg\right)=\left(cO_{2start\;}\cdot V_{start}\right)-\left(cO_{2end}\;\cdot V_{end}\right)\left[\frac{µg\cdot \mathrm{mL}}{\mathrm{mL}}\right]$$

(3) $$cOxyHb_{\;=}\frac{mO_{2}\;}{1.916\;}\cdot \left(\frac{1}{V_{end\;}}\right)$$

with *cO*_2*start*_ and *cO*_2*end*_ being the concentrations of oxygen in the solution before and after the addition of K_3_[Fe(CN)_6_], respectively, *V_start_* and *V_end_* initial and final sample volume, *mO*_2_ the mass of oxygen, and 1.916 (mg/g) the mass of oxygen (mg), which is bound by one gram Hb.

### 3.5. Preparation and Characterization of Hemoglobin Microparticles (Hb-MP), Human Serum Albumin Microparticles (HSA-MP), and Methemoglobin Particles (MetHb-MP)

Hb-MP and HSA-MP were fabricated following a slightly modified CCD-method described by Xiong et al. [12,14,33]. Briefly, 20 mL Na_2_CO_3_ (0.25 M) and 10 mL MnCl_2_ (0.5M) containing 0.5 g/dL bovine Hb or HSA were rapidly mixed in a 100 mL beaker under vigorous stirring for 30 s at room temperature to produce the manganese carbonate protein-microparticles (MnCO_3_-Hb-MP, MnCO_3_-HSA-MP). The carbonate-protein particles were separated by centrifugation at 3000× *g*, 4 °C for 3 min followed by three washing steps with NaCl 0.9% solution. For crosslinking of the entrapped proteins, the MnCO_3_-Hb-MP were incubated in a 0.02% GA solution and the MnCO_3_-HSA-MP in 0.01%, 0.02%, and 0.1% GA solutions, respectively, for 1 h at room temperature. Thereafter the MnCO_3_ templates were dissolved with 0.2 M Na_2_-EDTA solution (pH 7.4). Finally, the particles were washed three times with 0.9% NaCl solution containing 0.2 g/dL HSA (10,000× *g*, 4 °C, 10 min) and resuspended in PBS pH 7.4. The packed particle volume (PPV) in the suspension was adjusted to 10%. The particle size was determined by dynamic light scattering (Zetasizer Nano ZS, Malvern Instruments Ltd., Malvern, UK). All measurements were performed in triplicate at 25 °C.

The total hemoglobin concentration in the Hb-MP was measured by a modified AHD-575 method as described before [12]. Briefly, Hb-MP suspensions (PPV 2%) were digested by 0.5 mg/mL Pronase (Roche Diagnostics GmbH, Mannheim, Germany) at 45 °C for 30 min. Then AHD reagent was added (volume ratio 1:1) and incubated for further 15 min at room temperature. Finally, the samples were centrifuged (10,000× *g* for 10 min), the supernatants were collected, and their absorption was measured using a UV-VIS spectrophotometer at 575 nm (Hitachi U2800, Hitachi High-Technologies Corporation).

The total iron content (cFe) of the Hb-MP was measured by inductively coupled plasma optical emission spectroscopy (ICP-OES; Thermo, iCAP 6300 Duo, Thermo Fisher Scientific GmbH, Berlin, Germany). Before measurement the particles were dissolved with 6 M HCl.

The cOxyHb concentration in the Hb-MP was determined indirectly by the oxygen release method as described above for the RBC suspensions.

MetHb-MP were prepared as follows: Hb-MP suspensions (PPV 10%) were incubated with 5 mM NaNO_2_ solution in PBS pH 7.4 for 30 min under gentle mixing at room temperature (22 °C) and stored overnight at 4 °C. The MetHb-MP were washed three times with PBS (pH 7.4) and finally resuspended in PBS to a PPV of 10%.

### 3.6. NMR Analysis

For the relaxivity measurements, Hb and MetHb solution samples were prepared at cHb of 30 mg/mL in PBS pH 7.4. Different MetHb content was obtained by mixing Hb solution with MetHb solution at the ratios 100/0, 80/20, 60/40, 40/60, 20/80, and 0/100. All mixed Hb samples were diluted with PBS pH 7.4 to obtain solutions with different cHb and cMetHb.

The Hct of fresh RBC and MetHb-RBC suspensions was adjusted to 10% and then these suspensions were mixed at ratios 100/0, 80/20, 60/40, 40/60, 20/80, and 0/100 to obtain suspensions with different MetHb concentrations. Each mixed RBC sample was diluted with PBS pH 7.4 to Hct of 2% and 5%. GA-RBCs prepared with the different GA concentrations listed above were also diluted to Hct of 2% and 5% with PBS pH 7.4.

The Hb-MP and MetHb-MP suspensions (PPV 10%) were mixed at ratios 100/0, 80/20, 60/40, 40/60, 20/80, and 0/100 to obtain different MetHb concentrations in the particle suspensions. Then all mixed samples were diluted with PBS pH 7.4 to obtain suspensions with PPV of 2%, 4%, and 6%. HSA-MP cross-linked with different GA concentrations were also suspended in PBS pH 7.4 to PPV of 2%, 4%, and 6%.

A total of 1 mL of each sample was transferred into nuclear magnetic resonance (NMR) glass tube (7.5 mm inner diameter). Longitudinal relaxation time (T_1_) and transverse relaxation time (T_2_) were obtained by using a 0.94 T Minispec mq40 relaxometer (Bruker Analytik, Rheinstetten, Germany) operated at a proton frequency of 40 MHz and a preset temperature of 37 °C. The relaxation rates 1/T_1_ or 1/T_2_ were plotted versus concentration of the samples. The relaxivities *(r*_1_, *r*_2_) were obtained from the slope of the linear regression of the relaxation rate plot (1/*T_i_*; *i* = 1,2) versus concentration of investigated compound.

(4) $$r_{i\;}=\left[\;\frac{1}{T_{i}}-\;\frac{1}{T_{i}^{0}}\;\right]\frac{1}{c}\;;\;\;\;\;i=1,2$$

with *c* being the concentration, *T_i_*^0^ the relaxation time of the solvent without samples, and *T_i_* the longitudinal (*i* = 1) or transverse (*i* = 2) relaxation time of the samples.

## Figures and Tables

**Table 1:** Overview on the protein particles used in the ^1^H_2_O NMR experiments (N = 6).

			Values for Particle Suspensions with 20% PPV			
Type of MP	cGA	Size	cHb (by AHD)	cFe (ICP-OES)	cOxyHb (O_2_ Release)	cMetHb (cHb—cOxyHb)
	%	nm	mg/mL	mg/L	mg/mL	mg/mL
Hb-MP	0.02	804.5 ± 15.6	22.9 ± 3.3	77.4 ± 2.9	11.4 ± 0.5	11.5 ± 3.4
MetHb-MP	0.02	809.2 ± 15.4	24.3 ± 3.1	82.5 ± 4.7	0.9 ± 0.3	23.4 ± 5.0
HSA-MP	0.01	803.6 ± 65.5	-	-	-	-
HSA-MP	0.02	776.2 ± 47.0	-	-	-	-
HSA-MP	0.10	823.6 ± 28.9	-	-	-	-

## Supplementary Materials

Supplementary Materials can be found at https://www.mdpi.com/1422-0067/21/23/8978/s1. Figure S1: OxyHb concentration in Hb-solutions at various MetHb concentration (total Hb concentration, cHb, 30mg/mL in all solutions, orange squares) and in mixtures of fresh RBC with MetHb-RBC suspensions (Hct 10% in all suspensions, blue circles) determined by oxygen release method and blood gas analyzer (N = 6); Figure S2: Relaxation rates of human and bovine RBC (vital and GA-cross-linked) in dependency on the total Hb concentration.
