# Supplementary material for: Determination of Methemoglobin in Hemoglobin Submicron Particles Using NMR Relaxometry
Source: Int J Mol Sci. 2020 Nov 26;21(23):8978. doi: 10.3390/ijms21238978 (PMC7730817; doi:10.3390/ijms21238978)
Supplement: Supplementary file 1 [file ijms-21-08978-s001.pdf]

International Journal of
Molecular Sciences

MDPI

Supplementary

Determination of Methemoglobin in Hemoglobin Submicron Particles Using NMR Relaxometry

Waraporn Kaewprayoon [1,2], Nittiya Suwannasom [1,3], Chiraphat Kloypan [1,4], Axel Steffen [1], Yu Xiong [1], Eyk Schellenberger [5], Axel Pruß [1], Radostina Georgieva [1,6] and Hans Bäumler [1,*]

1 Charité-Universitätsmedizin Berlin, Institute of Transfusion Medicine, 10117 Berlin, Germany; waraporn.kpy@gmail.com (W.K.); nittiya.su@up.ac.th (N.S.); chiraphat.kl@up.ac.th (C.K.); Axel.steffen@charite.de (A.S.); Yu.Xiong@charite.de (Y.X.); axel.pruss@charite.de (A.P.); radostina.georgieva@charite.de (R.G.)
2 Department of Pharmacy, Payap University, Chiang Mai 50000, Thailand
3 Division of Biochemistry and Nutrition, School of Medical Sciences, University of Phayao, Phayao 56000, Thailand
4 Division of Clinical Immunology and Transfusion Sciences, School of Allied Health Sciences, University of Phayao, Phayao 56000, Thailand
5 Charité-Universitätsmedizin Berlin, Institute of Radiology and Children Radiology, 10117 Berlin, Germany; eyk.schellenberger@charite.de
6 Department of Medical Physics, Biophysics and Radiology, Faculty of Medicine, Trakia University, 6000 Stara Zagora, Bulgaria
* Correspondence: hans.baeumler@charite.de; Tel.: +49 (30) 450525131

Received: 30 October 2020; Accepted: 23 November 2020; Published: 26 November 2020

1. Comparison of oxyhemoglobin concentration (cOxyHb) measured by the oxygen release method (OR) and blood gas analyzer (ABL700)

The direct measurement of the OxyHb and MetHb contents in HbMP by means of a blood gas analyzer (multi-wave spectrophotometry) is not possible due to strong scattering of the particles. Therefore, the OxyHb concentrations (cOxyHb) of Hb-solutions and RBC suspensions containing different amounts of MetHb were measured in parallel by both, the oxygen release method (OR) and ABL700, and the obtained results were plotted against each other (Figure S1). The values for cOxyHb obtained by OR corresponded well to those measured by ABL700.

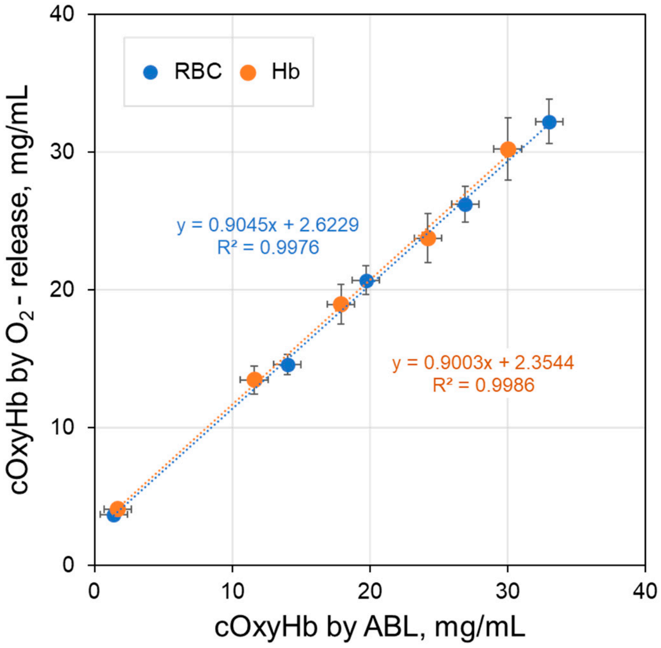

Figure 1. OxyHb concentration in Hb-solutions at various MetHb concentration (total Hb concentration, cHb, 30mg/mL in all solutions, orange squares) and in mixtures of fresh RBC with MetHb-RBC suspensions (Hct 10% in all suspensions, blue circles) determined by oxygen release method and blood gas analyzer (N=6). The standard deviations for low concentrations are too small to be visible.

2. Comparison of ^1H_2O NMR relaxation rates of vital and glutaraldehyde cross-linked human and bovine red blood cells (RBC)

H2O relaxation of human and bovine RBC)

The ^1H_2O NMR longitudinal ($1/T_1$) and transverse ($1/T_2$) relaxation rates of human and bovine RBC (native and cross-linked with GA-concentration 2%) were measured in suspensions with different hematocrit and displayed in Figure S2 in dependency on the total hemoglobin concentration (cHb). The values obtained for vital human and vital bovine RBC are practically equal, which was expected because bovine and human Hb have a very similar primary and secondary structure.

Both relaxations are strongly accelerated after GA cross-linking (GA-concentration 2%) due to the partial oxidation of the Hb to MetHb, conformational changes of the protein and higher accessibility of the iron for ^1H_2O. The relaxation rates measured for the bovine GA-RBC are higher than that of the human GA-RBC. This is due to the significantly smaller size of the bovine RBC (diameter 5 - 6 µm, average cell volume 40-60 fL [1] compared to the human RBC (diameter 7.5 - 8.5 µm, average cell volume 90 fL). GA-RBC are not deformable and at the same number of cells per volume the measured Hct is higher than the hematocrit of suspensions of native RBC, due to the different package of the cells upon centrifugation. The smaller bovine GA-RBC form a denser package

than the larger human GA-RBC and therefore contain a larger amount of Hb, which reflects in higher relaxation rates.

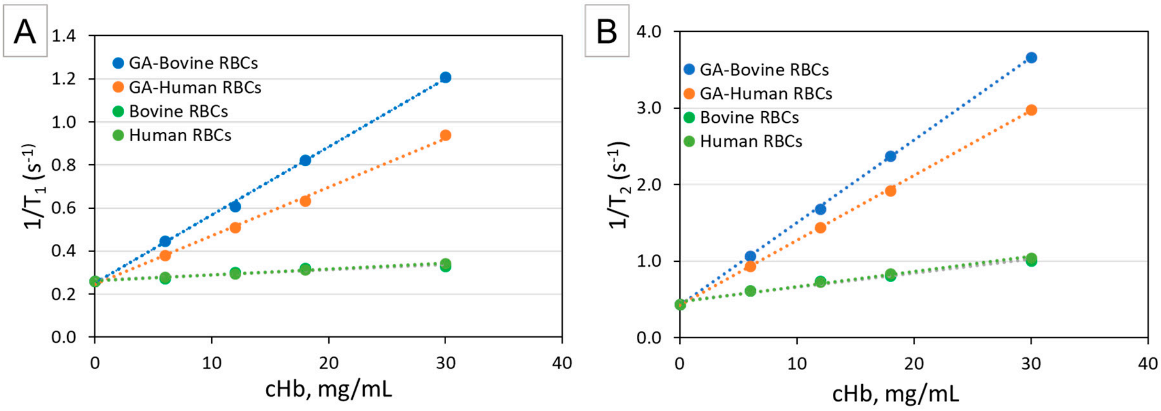

Regression: 1/T1 = A x cHb + B			
	A	B	R²
Human RBCs	0.0027	0.2628	0.9959
Bovine RBCs	0.0025	0.2647	0.9016
GA-Human RBCs	0.0226	0.2469	0.9969
GA-Bovine RBCs	0.0317	0.2524	0.9986

Regression: 1/T2 = A x cHb + B			
	A	B	R²
Human RBCs	0.3183	0.4733	0.9876
Bovine RBCs	0.2942	0.4821	0.9755
GA-Human RBCs	1.3623	0.4307	0.9996
GA-Bovine RBCs	1.7359	0.4267	0.9998

Figure 2. Relaxation rates of human and bovine RBC (vital and GA-cross-linked) in dependency on the total Hb concentration. (**A**) Longitudinal relaxation rate ($1/T_1$) and (**B**) transverse relaxation rate ($1/T_2$). GA cross-linking was performed with 2% GA (w/v).

References:

[1] W. Klee und I. Hartmann, Klinische Labordiagnostik in der Buiatrik 2017-07-02; http://www.rinderskript.net/skripten/Laborskript.pdf
